# Biogenic and Synthetic Polyamines Bind Cationic Dendrimers

**DOI:** 10.1371/journal.pone.0036087

**Published:** 2012-04-27

**Authors:** Jean-Sebastian Mandeville, Phillipe Bourassa, Thekkumkattil John Thomas, Heidar-Ali Tajmir-Riahi

**Affiliations:** 1 Département de Chimie-Biologie, Université du Québec à Trois-Rivières, Trois-Rivières, Québec, Canada; 2 Department of Medicine, University of Medicine and Dentistry of New Jersey-Robert Wood Johnson Medical School, New Brunswick, New Jersey, United States of America; 3 The Cancer Institute of New Jersey, University of Medicine and Dentistry of New Jersey, Robert Wood Johnson Medical School, New Brunswick, New Jersey, United States of America; Dalhousie University, Canada

## Abstract

Biogenic polyamines are essential for cell growth and differentiation, while polyamine analogues exert antitumor activity in multiple experimental model systems, including breast and lung cancer. Dendrimers are widely used for drug delivery *in vitro* and *in vivo*. We report the bindings of biogenic polyamines, spermine (spm), and spermidine (spmd), and their synthetic analogues, 3,7,11,15-tetrazaheptadecane.4HCl (BE-333) and 3,7,11,15,19-pentazahenicosane.5HCl (BE-3333) to dendrimers of different compositions, mPEG-PAMAM (G3), mPEG-PAMAM (G4) and PAMAM (G4). FTIR and UV-visible spectroscopic methods as well as molecular modeling were used to analyze polyamine binding mode, the binding constant and the effects of polyamine complexation on dendrimer stability and conformation. Structural analysis showed that polyamines bound dendrimers through both hydrophobic and hydrophilic contacts with overall binding constants of *K*_spm-mPEG-G3_ = 7.6×10^4^ M^−1^, *K*_spm-mPEG-PAMAM-G4_ = 4.6×10^4^ M^−1^, K_spm-PAMAM-G4_ = 6.6×10^4^ M^−1^, *K*_spmd-mPEG-G3_ = 1.0×10^5^ M^−1^, *K*_spmd-mPEG-PAMAM-G4_ = 5.5×10^4^ M^−1^, K_spmd-PAMAM-G4_ = 9.2×10^4^ M^−1^, *K*_BE-333-mPEG-G3_ = 4.2×10^4^ M^−1^, *K*_Be-333-mPEG-PAMAM-G4_ = 3.2×10^4^ M^−1^, K_BE-333-PAMAM-G4_ = 3.6×10^4^ M^−1^, *K*_BE-3333-mPEG-G3_ = 2.2×10^4^ M^−1^, *K*_Be-3333-mPEG-PAMAM-G4_ = 2.4×10^4^ M^−1^, K_BE-3333-PAMAM-G4_ = 2.3×10^4^ M^−1^. Biogenic polyamines showed stronger affinity toward dendrimers than those of synthetic polyamines, while weaker interaction was observed as polyamine cationic charges increased. The free binding energies calculated from docking studies were: −3.2 (spermine), −3.5 (spermidine) and −3.03 (BE-3333) kcal/mol, with the following order of binding affinity: spermidine-PAMAM-G-4>spermine-PAMMAM-G4>BE-3333-PAMAM-G4 consistent with spectroscopic data. Our results suggest that dendrimers can act as carrier vehicles for delivering antitumor polyamine analogues to target tissues.

## Introduction

**Figure 1:**
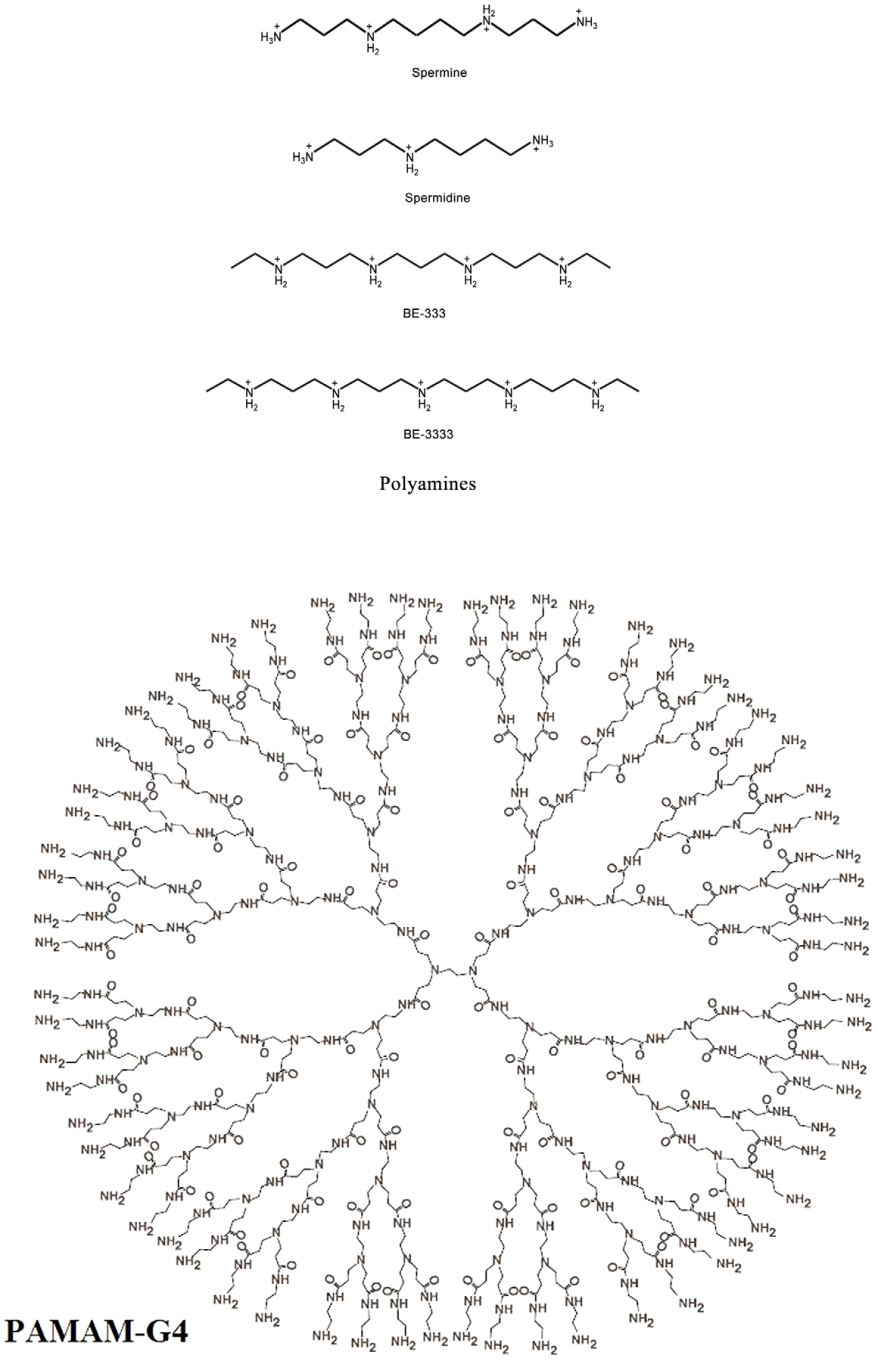
Chemical structures of polyamines and PAMAM-G4 dendrimer.

Polyamine analogues (Fig. 1) exert antitumor activity in multiple experimental model systems, including breast and lung cancer models and they are being used in clinical trials [1]–[5]. Synthetic polyamines can mimic some of the self-regulatory functions of biogenic polyamines but are unable to substitute for natural polyamines in their growth promoting role [6]–[13]. Natural polyamines are ubiquitous cellular cations and are involved in cell growth and differentiation (14). They are capable of modulating gene expression and enzyme activities, activation of DNA synthesis, and facilitating protein-DNA interactions [13]–[20]. Even though interactions of biogenic and synthetic polyamines with DNA and RNA are well characterized [21]–[25], little is known about their interaction with therapeutically important synthetic polymers, such as dendrimers [26].

Synthetic polymers with a specific shape and size play important roles in the development of modern drug and gene delivery systems [27]–[29]. Dendrimers are unique synthetic macromolecules of nanometer dimensions with a highly branched structure and globular shape [29], [30]. Among dendrimers, polyamidoamine (PAMAM) dendrimers (Fig. 1) have received most attention as potential gene and drug delivery systems [31]–[33]. Several attempts have been made to design different dendrimers as drug carriers [34]. For example, anticancer fluorouracil drug was attached to dendrimers with a cyclic core [35], while dendrimers with poly(ethylene glycol) grafts were used to encapsulate antitumor drugs adriamycin and methotrexate [36]. Similarly, it has been shown that a poly(propyleneimine) dendrimers endcapped with 64 L-phenylalanine encapsulated nearly 4 molecules of Bengal Rose for every dendritic molecule [37]. Since dendrimers have a large number of terminal groups to which drug molecules can be attached, they can carry drug molecules with a high efficiency. They contain several binding sites for hydrophobic, hydrophilic, cationic and anionic drugs. In developing dendrimers for drug delivery, it is important to use dendrimers with low toxicity and excellent biocompatibility. However, dendrimers such as PAMAM (polyamidoamine) and polypropyleneimine (PPI) are toxic. It has been demonstrated that modification of the amino groups on the periphery of dendrimers with poly(ethylene glycol) could reduce toxicity and increase biocompatibility [38]–[40]. Poly(ethylene glycol) is nontoxic, nonimmunogenic and water soluble, and its combination with other substrates produces conjugated molecules, that combine the properties of both the substrate and the polymer. However, conjugate formation can alter the binding affinity of dendrimers in general since a part of the functional pendant groups are removed by conjugation.

In this report, we present the results of spectroscopic and molecular docking experiments on the interaction of biogenic and synthetic polyamines with dendrimers of different composition, PAMAM (G4), m-PEG-PAMAM (G3) and m-PEG-PAMAM (G4), in aqueous solution, using a constant polymer concentration and different drugs concentrations. Structural data regarding polyamine binding modes as well as the stability of polyamine-dendrimer complexes are presented in this report.

## Materials and Methods

### Materials

Spermine.4HCl and spermidine.3HCl were purchased from Sigma Chemical Company and used as supplied. Polyamine analogues, BE-333 and BE-3333, were synthesized in the laboratory of Dr. Akira Shirahata (Josai University, Saitama, Japan). PAMAM-G4 (MW 14214 g/mol) was purchased from Aldrich Chemical Co and used as supplied. mPEG-PAMAM-G3 (MW 13423 g/mol) and mPEG-PAMAM-G4 (MW 19214 g/mol) were synthesized according to published methods [35], [41], [42]. mPEG block has a molecular weight of 5000 g/mol. Other chemicals were of reagent grade and used without further purification.

### Preparation of stock solutions

Dendrimer solution (1 mM) were prepared in distilled water and diluted to various concentrations in Tris-HCl buffer. Polyamine solutions (1 mM) were prepared in water and diluted in Tris-HCl buffer. The pH of stock solutions was kept at 7±0.2.

### FTIR spectroscopic measurements

Infrared spectra were recorded on a FTIR spectrometer (Impact 420 model), equipped with deuterated triglycine sulphate (DTGS) detector and KBr beam splitter, using AgBr windows. Polyamine solutions were added drop-wise to dendrimer solutions, with constant stirring to ensure the formation of homogeneous solutions and to reach target polyamine concentrations of 0.125, 0.25, and 0.5 mM and a final dendrimer concentration of 0.5 mM. Spectra were collected after 2 h incubation of polyamine and polymer solution at room temperature, using hydrated films [42]. Interferograms were accumulated over the spectral range of 4000-600 cm^−1^, with a nominal resolution of 4 cm^−1^ and 100 scans. The difference spectra [(dendrimer+polyamine solution)−(dendrimer solution)] were generated, using dendrimer bands at 843 (mPEG-PAMAM-G3), 841 (mPEG-PAMAM-G4) and 1037 cm^−1^ (PAMAM-G4). These vibrations are related to the polymers C-C stretching and semi ring skeletal modes [43], [44] that show no spectral changes (intensity or shifting) upon polyamine-dendrimer complex formation, and cancelled on spectral subtraction.

### UV-Visible spectroscopy

The UV-Vis spectra were recorded on a Perkin-Elmer Lambda spectrophotometer with a slit of 2 nm and scan speed of 400 nm min^−1^. Quartz cuvettes of 1 cm were used. The absorbance measurements were performed at pH 7.0 by keeping the concentration of dendrimer constant (0.10 mM), while increasing polyamine concentrations (0.005 mM to 0.10 mM).

The binding constants were obtained according to the method described by Connors [45]. It is assumed that the interaction between the ligand L and the substrate S is 1∶1; for this reason a single complex SL (1∶1) is formed. It was also assumed that the sites (and all the binding sites) are independent and all species obeyed the Beer's law. A wavelength is selected at which the molar absorptivities ε_S_ (molar absorptivity of the substrate) and ε_11_ (molar absorptivity of the complex) are different. In the absence of ligands and light path length (b) of 1 cm and at total substrate concentration S_t_, the solution absorbance is given by the following equation:
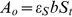
(1)At total concentration L_t_ of a ligand, the absorbance of a solution containing the same total substrate concentration is:
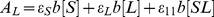
(2)where [S] is the concentration of the uncomplexed substrate, [L] the concentration of the uncomplexed ligand and [SL] is the concentration of the complex) which, combined with the mass balance on S and L, gives
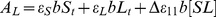
(3)where Δ*ε_11_ = ε_11_−ε_S_−ε_L_* (*ε_L_* molar absorptivity of the ligand). By measuring the solution absorbance against a reference containing ligand at the same total concentration L_t_, the measured absorbance becomes
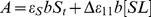
(4)Combining equation (4) with the stability constant definition K_11_ = [SL]/[S][L], gives
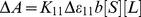
(5)where Δ*A = A−A_o_* . From the mass balance expression S_t_ = [S]+[SL] we get [S] = S_t_/(1+*K*_11_[L]), which is equation (5), giving equation (6) at the relationship between the observed absorbance change per centimeter and the system variables and parameters.
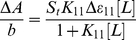
(6)Equation (6) is the binding isotherm, which shows the hyperbolic dependence on free ligand concentration.

The double-reciprocal form of plotting the rectangular hyperbola 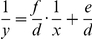, is based on the linearization of equation (6) according to the following equation,
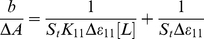
(7)


Thus the double reciprocal plot of 1/ΔA versus 1/[L] is linear and the binding constant can be estimated from the following equation
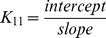
(8)


### Molecular modeling

The PAMAM-G4 and polyamine structures were generated using the ChemOffice Ultra 6.0 software suite. The polyamine was then automatically docked to the rough PAMAM-G4 structure using ArgusLab 4.0.1 (ArgusLab 4.0.1, Mark A. Thompson, Planaria Software LLC, Seattle,WA, http://www.arguslab.com). The docked polyamine-PAMAM-G4 structures were optimized by means of molecular dynamics using the MM+ force field available in HyperChem Pro 7.0. The heat time and run time for the simulations were 2 ps and 28 ps respectively with a step size of 0.001 ps. The temperature was initially set at 1 K and gradually increased to 300 K during the heat time by increments of 30 K. In all the simulations, equilibrium (achieving constant temperature near the selected final value) was reached after approximately 20 ps. The free binding energies of the optimized PAMAM-G4–polyamine complex structures were calculated using the Ascore scoring function provided in the ArgusLab software.

## Results

### FTIR spectral analysis of polyamine-dendrimer complexes

**Figure 2:**
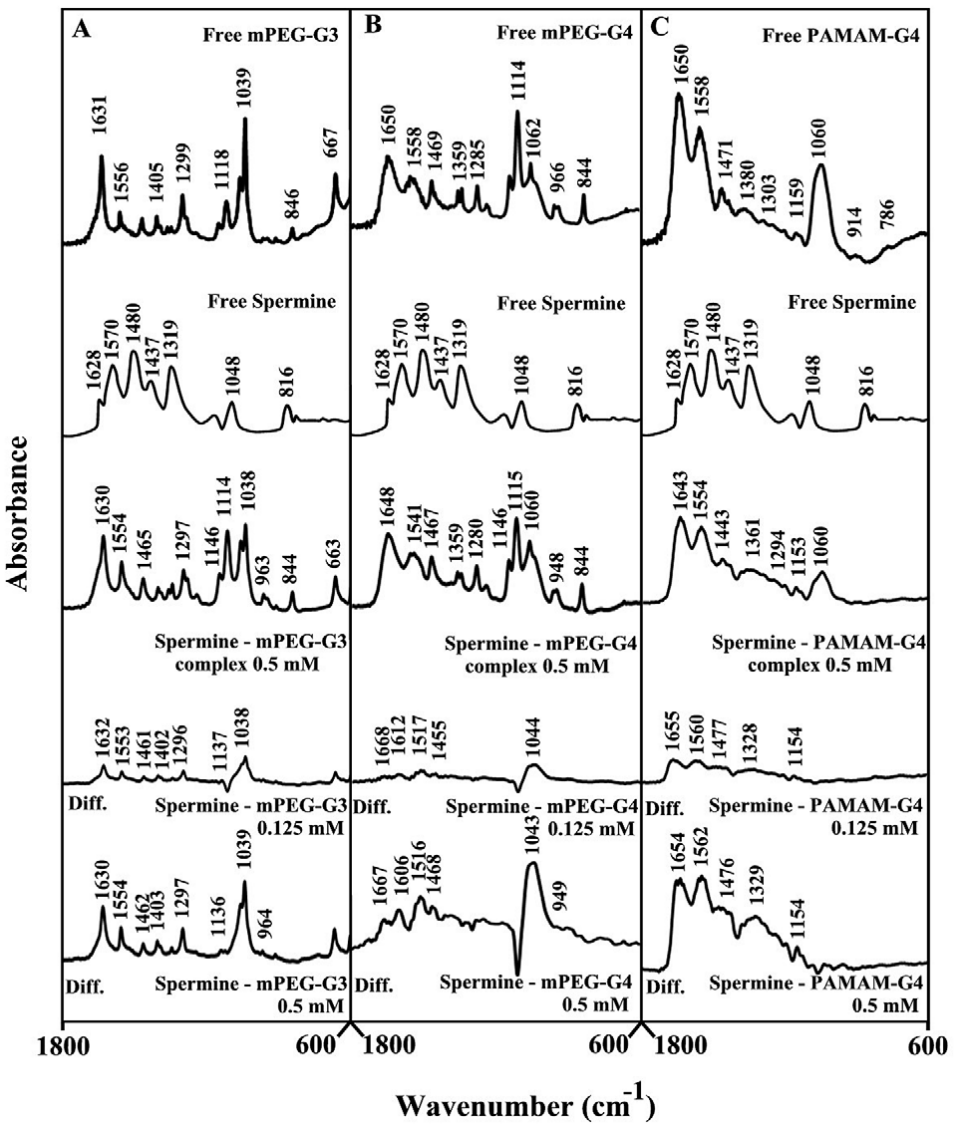
FTIR spectra and difference spectra (diff.) in the region of 1800-600 cm^−1^ of hydrated films (pH 7.4) for free mPEG-PAMAM-G3 (A), mPEG-PAMAM-G4 (B) PAMAM-G4 (C) (0.5 mM) and their spermine complexes obtained at different spermine concentrations (indicated on the figure).

Figure 2 shows the infrared spectra and difference spectra of dendrimers complexed with spermine. Spectral shifting was observed for the polymer C = O, C-N, C-O stretching and NH bending [43], [44] due to drug hydrophilic interactions with polymer polar groups. The major infrared bands at 1631 (C = O stretch and NH bending), 1556 (C-N stretch), 1405, 1299 (C-O), 1118, 1061 and 1039 cm^−1^ (C-O and C-C stretch), in the infrared spectra of the free mPEG-PAMAM-G3 exhibited shifting and intensity increases upon spermine binding (Figs. 2A). Similarly, the major infrared bands of the free mPEG-PAMAM-G4 at 1650, 1558, 1469, 1359, 1285, 1114 and 1062 cm^−1^ showed shifting and intensity changes upon complex formation with spermine (Fig. 2 B). The infrared bands of the free PAMAM-G4 at 1650, 1558, 1471, 1380, 1159 and 1060 cm^−1^ also shifted upon spermine interaction (Fig. 2C). The observed spectral shifting was accompanied with gradual increase in intensity of the above vibrational frequencies in the difference spectra [(dendrimer+spermine solution)−(dendrimer solution)] of drug-polymer complexes (Fig. 2A, 2B and 2C, diffs). The spectral changes observed are attributed to the hydrophilic interactions of polyamine polar groups with dendrimer NH_2_, C-O and C-N groups. The hydrophilic interaction is more pronounced at high spermine concentrations as evidenced by an increase in the intensity of several positive bands, centered at 1650-1000 cm^−1^ in the difference spectra of polyamine-dendrimer complexes (Fig. 2A, 2B and 2C, compare diffs 0.125 and 0.5 mM).

**Figure 3:**
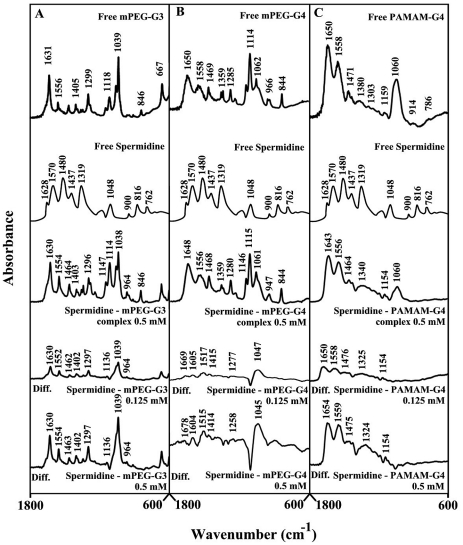
FTIR spectra and difference spectra (diff.) in the region of 1800-600 cm^−1^ of hydrated films (pH 7.4) for free mPEG-PAMAM-G3 (A), mPEG-PAMAM-G4 (B) PAMAM-G4 (C) (0.5 mM) and their spermidine complexes obtained at different spermidine concentrations (indicated on the figure).

Spermidine-polymer complex formation produced major spectral changes of the dendrimer infrared vibrational frequencies (Fig. 3). The spectral changes were observed mainly for the C = O, C-N, C-O stretching and NH bending modes [43], [44] in the region of 1650-1000 cm^−1^ of the infrared spectra of mPEG-PAMAM-G3, mPEG-PAMAM-G4 and PAMAM-G4, upon complex formation with spermidine (Fig. 3A, 3B and 3C). The spectral shifting was associated with an increase in intensity of these vibrations in the difference spectra of spermidine-dendrimer complexes (Fig. 3A, 3B and 3C, diffs). More perturbations of polymer spectra occurred at high polyamine concentrations, as the intensity of the positive features increased as a result of spermidine-polymer complex formation (Fig. 3A, 3B and 3C. Compare diffs of 0.125 mM and 0.50 mM). The observed spectral changes are attributed to the hydrophilic contacts of drug OH groups with dendrimer NH_2_, C-O and C-N groups.

**Figure 4:**
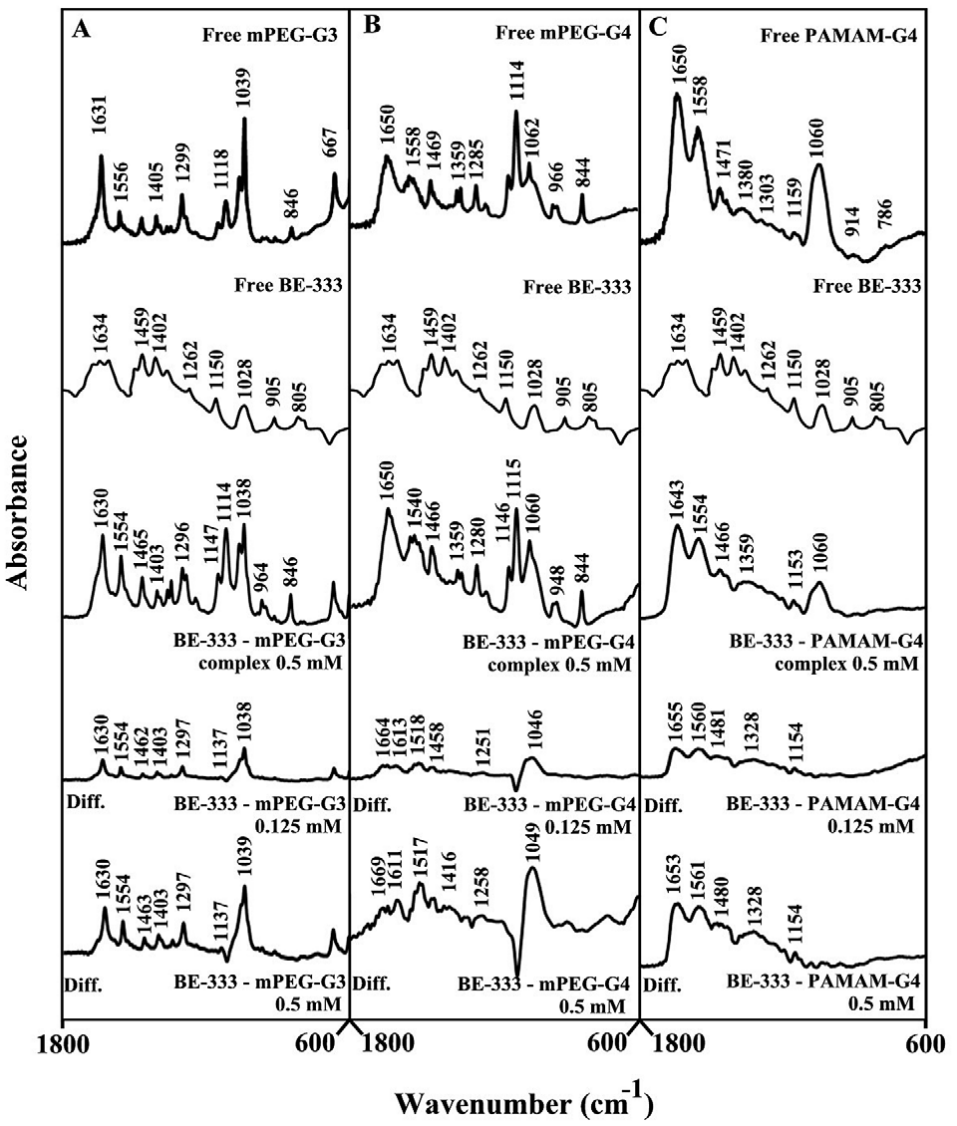
FTIR spectra and difference spectra (diff.) in the region of 1800-600 cm^−1^ of hydrated films (pH 7.4) for free mPEG-PAMAM-G3 (A), mPEG-PAMAM-G4 (B) PAMAM-G4 (C) (0.5 mM) and their BE-333 complexes obtained at different BE-333 concentrations (indicated on the figure).

BE-333-dendrimer complexation caused minor spectral changes (shifting and intensity) at low polyamine analogue concentration, while major spectral changes occurred at high polyamine concentrations (Fig. 4A, 4B and 4C, 0.125 and 0.5 mM). The observed spectral changes (shifting and intensity increases) are due to BE-333-polymer complex formation *via* dendrimer C-O, C-N and NH_2_ and the polyamine NH_2_ groups.

**Figure 5:**
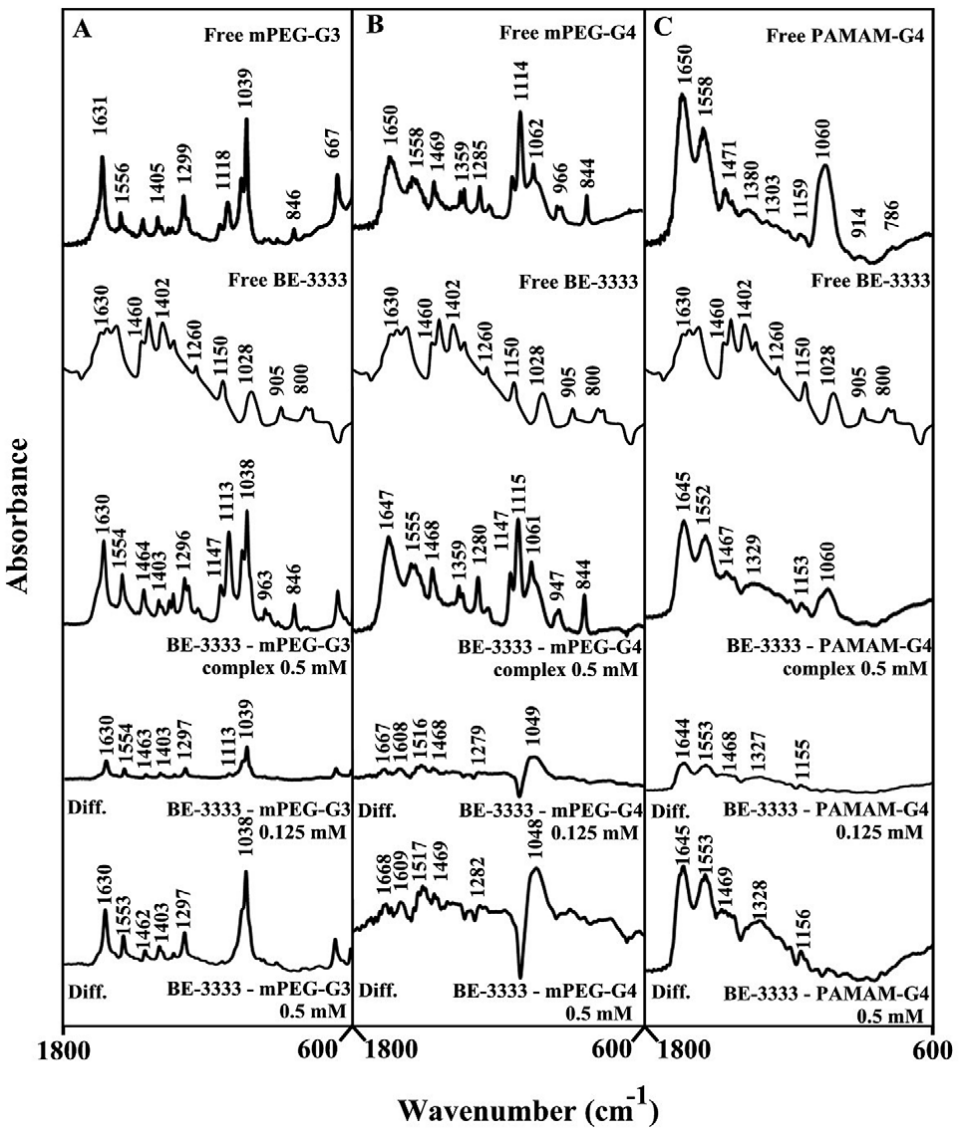
FTIR spectra and difference spectra (diff.) in the region of 1800-600 cm^−1^ of hydrated films (pH 7.4) for free mPEG-PAMAM-G3 (A), mPEG-PAMAM-G4 (B) PAMAM-G4 (C) (0.5 mM) and their BE-3333 complexes obtained at different BE-3333 concentrations (indicated on the figure).

The infrared spectra of BE-3333-dendrimer complexes presented in Fig. 5 showed minor spectral changes at low polyamine concentration and major shifting and intensity changes at high BE-3333 concentrations (Fig. 5A, 5B, 5C, compare 0.125 and 0.5 mM). The observed spectral changes are due to polyamine-polymer interaction *via* dendrimer C-O, C-N and NH and polyamine NH_2_ groups (hydrophilic contacts).

### Hydrophobic contacts

**Figure 6:**
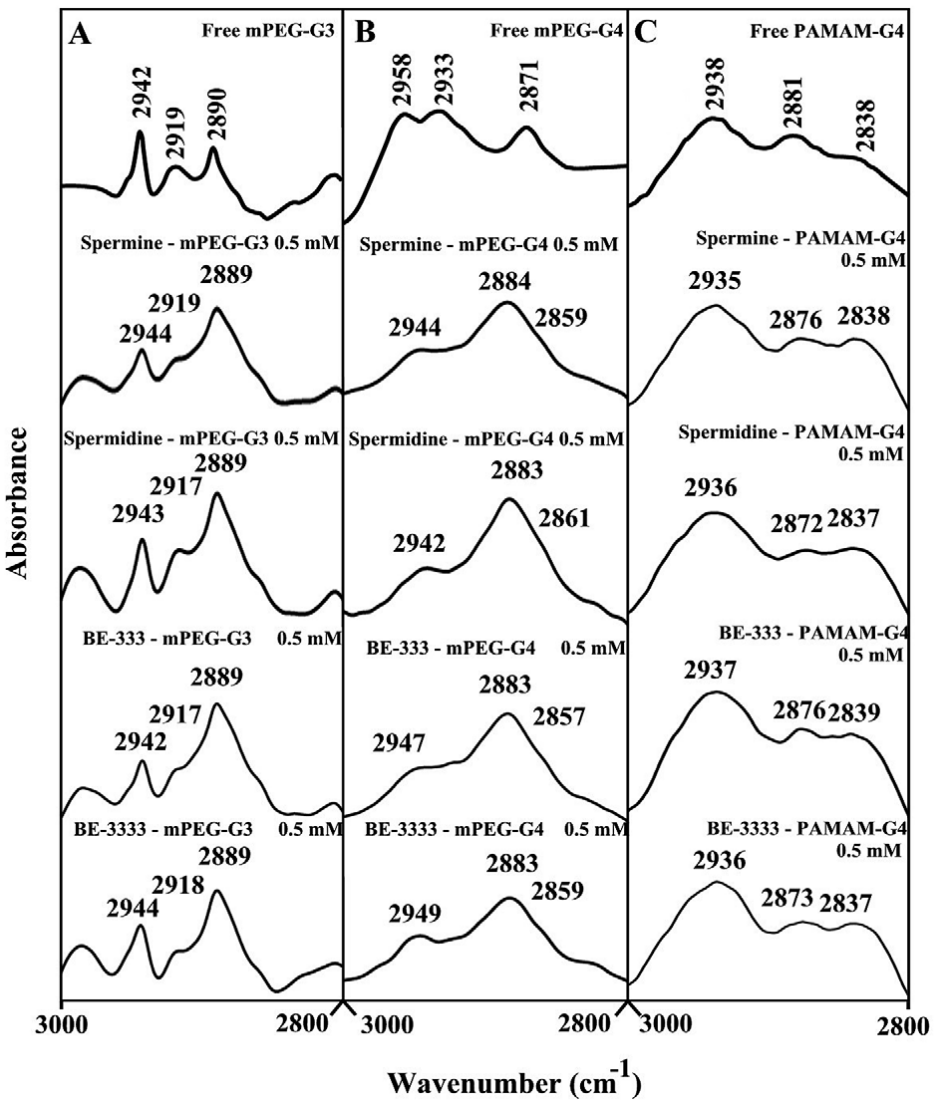
FTIR spectra in the region of 3300-2800 cm^−1^ of hydrated films (pH 7.4) for free mPEG-PAMAM-G3 (A), mPEG-PAMAM-G4 (B) and PAMAM-G4 (C) and their polyamine complexes obtained with 0.5 mM polymer and polyamine concentrations.

The effect of polyamine-polymer complex formation on dendrimer antisymmetric and symmetric CH_2_ stretching vibrations in the region of 3000-2800 cm^−1^ was investigated by infrared spectroscopy [43.44]. From Fig. 6A, the antisymmetric and symmetric CH_2_ bands of the free mPEG-PAMAM-G3 and its polyamine complexes were assigned as follows: free mPEG-PAMAM-G3 at 2942, 2912 and 2890 cm^−1^, spermine-mPEG-PAMAM-G3 at 2944 and 2889 cm^−1^; spermidine-mPEG-PAMAM-G3 at 2943, 2917 and 2889 cm^−1^; BE-333-mPEG-PAMAM-G3 at 2942, 2917 and 2889 cm^−1^; and BE-3333-mPEG-PAMAM-G3 at 2944, 2918 and 2889 cm^−1^. In Fig. 6B, the antisymmetric and symmetric CH_2_ bands of the free mPEG-PAMAM-G4 and its polyamine complexes were observed as follows: mPEG-PAMAM-G4, 2958, 2933 and 2817 cm^−1^; spermine-mPEG-PAMAM-G4, 2944, 2984 and 2859 cm^−1^; spermidine-mPEG-PAMAM-G4, 2942, 2983 and 2861 cm^−1^; BE-333-mPEG-PAMAM-G4, 2947, 2983 and 2857 cm^−1^; BE-3333-mPEG-PAMAM-G4, 2949, 2983 and 2859 cm^−1^. Similarly, Fig. 6C shows the CH_2_ stretching vibrations of the free PAMAM-G4 and its complexes as follows: PAMAM-G4, 2938, 2881 and 2838 cm^−1^; spermine-PAMAM-G4, 2935 and 2876 and 2838 cm^−1^; spermidine-PAMAM-G4, 2936, 2872 and 2837 cm^−1^; BE-333-PAMAM-G4, 2937, 2876 and 2839 cm^−1^; and BE-3333-PAMAM-G4, 2936, 2873 and 2837 cm^−1^. The observed spectral shifting for polymer CH_2_ vibrations is indicative of some degree of hydrophobic interactions for polyamine-dendrimer complexes. This is due to the hydrophobic contacts *via* polyamine hydrophobic parts (aliphatic CH_2_ groups) and the interior hydrophobic cavities present in dendrimers.

### UV-Visible spectra and stability of polyamine-dendrimer complexex

**Figure 7:**
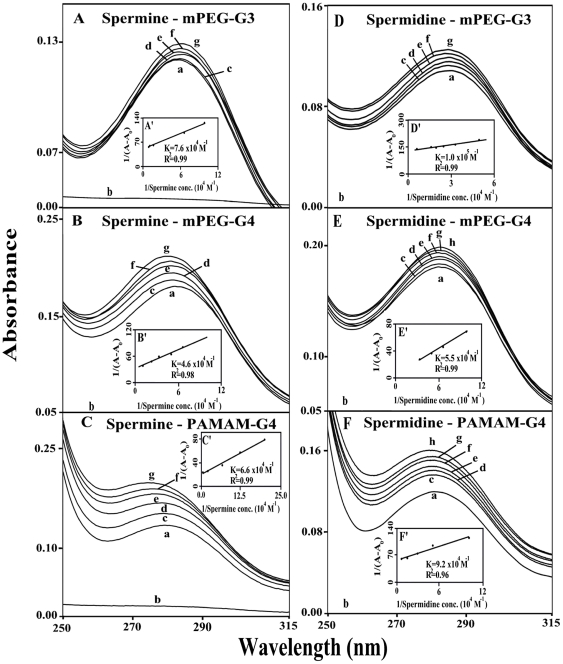
UV-visible spectra of mPEG-PAMAM-G3, mPEG-PAMAM-G4 and PAMAM-G4 and their complexes with spermine and spermidine with free dendrimer at 100 µM and complexes c-g at 5, 10, 20, 40 and 80 µM. **(A, B and C) for spermine and c-g at 5, 10, 20, 40, and 80 µM for spermidine.mPEG-G3 (D), c-h c-g at 5, 10, 20, 40, 80 and 100 µM for spermidine-mPEG-G4 (E) and spermidine-PAMAM-G4 (F). Plots of 1/(A−A0) vs (1/ polyamine concentration) and binding constant (** ***K*** **) for spermine (A′, B′ and C′) and spermidine (D′, E′ and F′).**

**Figure 8:**
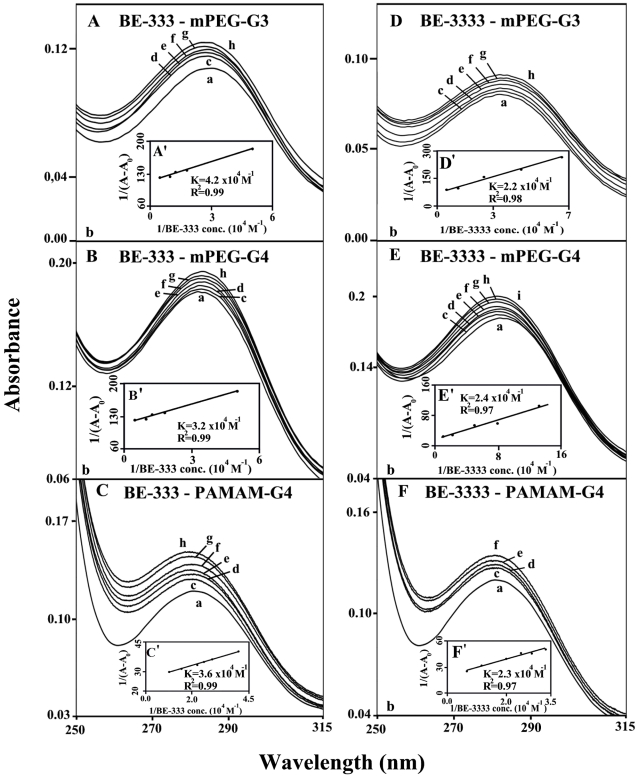
UV-visible spectra of mPEG-PAMAM-G3, mPEG-PAMAM-G4 and PAMAM-G4 and their complexes with BE-333 and BE-3333 with free dendrimer at 100 µM and complexes c-h at 5, 10, 20, 40, 80 and 100 µM. **(A, B and C); c-h at 5, 10, 20, 40, 80 and 100 µM for BE-3333-mPEG-G3 (D), c-i at at 5, 10, 20, 40, 60, 80 and 100 µM BE-3333-mPEG-G4 (E) and c-f at 5, 10, 20 and 40 µM ( F).** Plots of 1/(A−A0) vs (1/ polyamine concentration) and binding constant (*K*) for BE-333 (**A′**, **B′** and **C′**) and BE-3333 (**D′**, **E′** and **F′**).

The UV spectra of polyamine-dendrimer complexes are presented in Figures 7 (spermine and spermidine) and 8 (BE-333 and BE-3333). There is clear evidence that as polyamine complex formation occurred, major intensity increases of the dendrimer UV band, centered at 260–290 nm, also occurred [46], [47]. The spectral changes are more pronounced in the case of biogenic spermine and spermidine than those of polyamine analogues BE-333 and BE-3333 (Figs. 7 and 8).

The polyamine-dendrimer binding constants, obtained (according to the method described in experimental section (using plots of 1/(A−A_0_) vs (1/polyamine concentrations), showed one binding constant for each polyamine-polymer complex formation (Figs. 7 and 8 and Table 1). The calculated binding constants are: *K*_spm-mPEG-G3_ = 7.6×10^4^ M^−1^, *K*_spm-mPEG-PAMAM-G4_ = 4.6×10^4^ M^−1^, K_spm-PAMAM-G4_ = 6.6×10^4^ M^−1^, *K*_spmd-mPEG-G3_ = 1.0×10^5^ M^−1^, *K*_spmd-mPEG-PAMAM-G4_ = 5.5×10^4^ M^−1^, K_spmd-PAMAM-G4_ = 9.2×10^4^ M^−1^, *K*_BE-333-mPEG-G3_ = 4.2×10^4^ M^−1^, *K*_Be-333–mPEG-PAMAM-G4_ = 3.2×10^4^ M^−1^, K_BE-333-PAMAM-G4_ = 3.6×10^4^ M^−1^, *K*_BE-3333-mPEG-G3_ = 2.2×10^4^ M^−1^, *K*_Be-3333–mPEG-PAMAM-G4_ = 2.4×10^4^ M^−1^, K_BE-3333-PAMAM-G4_ = 2.3×10^4^ M^−1^ (Table 1). The binding affinity of biogenic polyamines toward dendrimers was stronger than that of synthetic polyamines, while weaker interaction was observed as polyamine cationic charge increased (Table 1). The reason why biogenic polyamine-dendrimers are more stable than those of the synthetic polyamines can be due to other factors such as the primary amines ((NH3+) in biogenic polyamines, that possess a higher density of positive charge than the secondary ones ((NH2+), in synthetic polyamines and also the presence of more hydrophobic contacts in the biogenic polyamine-polymer complexes.

**Table 1:** Binding constants of polyamine-dendrimers (*K* M^−1^).

Polyamines	mPEG-G3	mPEG-G4	PAMAM-G4
Spermine	(7.6±1)×10^4^	(4.6±0.6)×10^4^	(6.6±1)×10^4^
Spermidine	(1±0.4)×10^5^	(5.5±0.7)×10^4^	(9.2±1)×10^4^
BE-333	(4.2±0.6)×10^4^	(3.2±0.5)×10^4^	(3.6.±0.6)×10^4^
BE-3333	(2.2±0.5)×10^4^	(2.4±0.4)×10^4^	(2.3±0.4)×10^4^

### Docking studies

**Figure 9:**
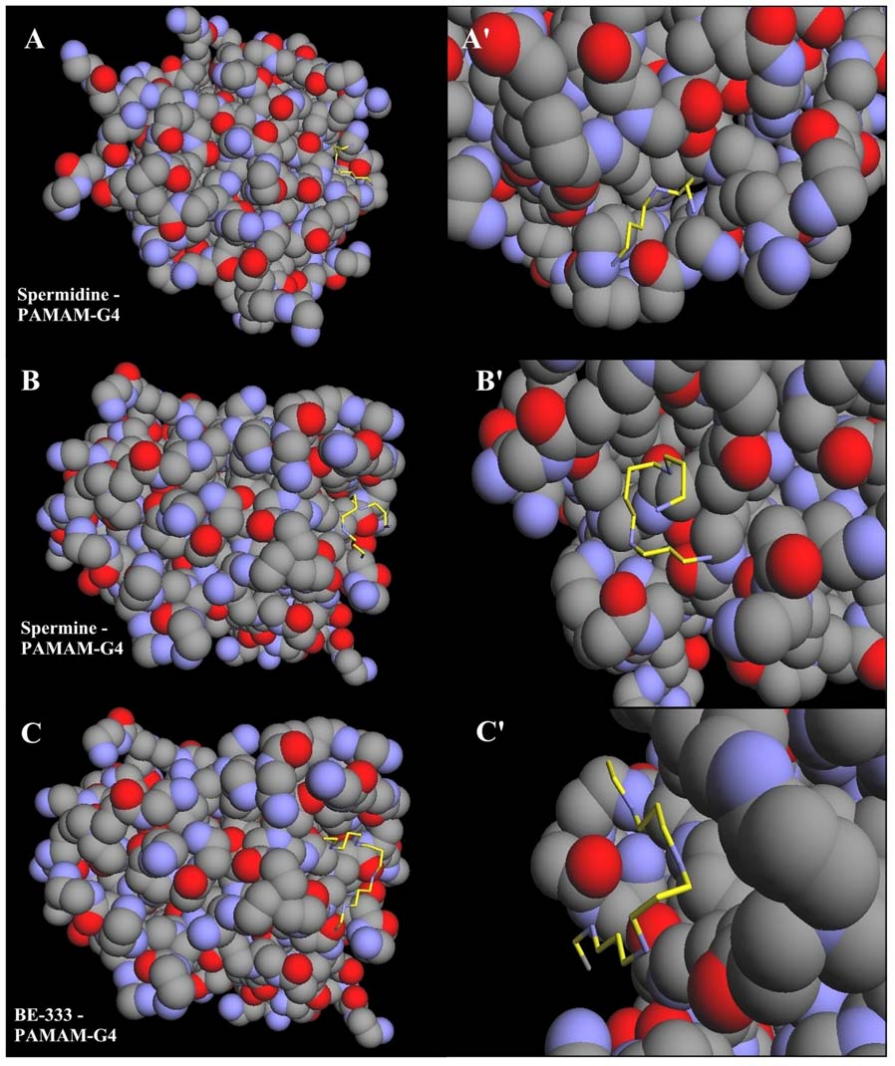
Optimized polyamine-PAMAM-G4 docking structures. The polyamines are shown in yellow color. (A) shows whole PAMAM-G4 in spheres with spermine and (A′) shows the zoom on the binding site represented in sticks. (B) shows whole PAMAM-G4 in spheres with spermidine and (B′) shows the binding site represented in sticks. (C) whole PAMAM-G4 in spheres with BE-333 and (C′) shows the binding site in represented in sticks.

Our results from FTIR and UV-visible spectroscopic methods were complemented with molecular dynamic simulations in which the polyamines spermine, spermidine and BE-333 were automatically docked to PAMAM-G4 and the resulting structures were optimized using the MM+ force field to determine the preferred conformations of the polyamine-polymer complexes. The simulation results are shown in Fig. 9 and Table 2. The models showed that polyamines are located on the surface of dendrimers and in cavities of PAMAM-G4 polymer (Fig. 9). The free binding energies calculated from docking studies were as follows: spermine, (3.2; spermidine, (3.5 and BE-333, (3.03 kcal/mol, with the following order of binding affinity: spermidine-PAMAM-G-4>spermine-PAMMAM-G4>BE-333-PAMAM-G4. These results are consistent with the data obtained from spectroscopic studies (Tables 1 and 2).

**Table 2:** Free binding energy of the docked polyamine-PAMAM complexes.

Complex	Δ*G_binding_* (kcal/mol)
Spermidine – PAMAM-G4	−3.50
Spermine – PAMAM-G4	−3.20
BE-333 – PAMAM-G4	−3.03

## Discussion

Several synthetic macromolecules have been developed as drug and gene delivery vehicles [27], [28], [30], [32]. An ideal drug carrier vehicle must be biochemically inert and non-toxic, while protecting the payload (drug) from dissociation until it reaches the target site, and capable of releasing the drug at target site. Among synthetic polymers, dendrimers are unique macromolecules with nanometer dimensions, a highly branched structure and globular shape. These macromolecules have uniform size and are mono-disperse, with modifiable surface functionality as well as internal cavities. They contain several binding sites for hydrophobic, hydrophilic, cationic and anionic drugs (Fig. 1) Dendrimers are capable of binding and transporting DNA, RNA and drug molecules with high efficiency [30], [32]. Dendrimers can be used as a containers to encapsulate drug molecules and carry them to different targets *in vivo* [35], [48], [49]. It has been shown that dendrimers with a hydrophobic interior and hydrophilic chain ends are capable of solubilizing hydrophobic compounds in aqueous solutions [49], [50], [51]. Attempts have been made to design different dendrimers as drug carriers [36]. For example, anticancer drug, 5-fluorouracil (5-FU) was attached to dendrimers with cyclic core [36]. Dendrimers having poly(ethylene glycol) grafts have been used to encapsulate antitumor drugs adriamycin and methotrexate [37]. The complexation of dendrimers with anti-inflammatrory drug flurbiprofen was studied *in vitro* and *in vivo*, while drug biodistribution in different organs has been monitored [39]. Gene delivery targeted to brain has been attempted using transferring-conjugated polyethyleneglycol-modified polyamidoamine dendrimer [40]. The purpose of our investigation was to analyze the interaction of dendrimers with biogenic and synthetic polyamines in order to test the feasibility of these nanocarrier molecules for polyamine-based drug delivery. Infrared spectroscopic data in the region of 1700-1000 cm^−1^, where most of the polymer in-plane vibrations related to C = O, C-N, NH and C-O modes are located, exhibit spectral changes (shifting and intensity variations) upon polyamine-polymer complex formation. These changes are more profound at high polyamine concentrations. There was clear evidence that the hydrophilic polyamine entity induced more perturbations of polymer hydrophilic group vibrational frequencies, with the following order of spectral changes: spermidine>spermine>BE-333>BE-3333 (Figs. 2, 3, 4, 5). This can be expected since polyamines with several positively charged NH and NH_2_ groups show more affinity for dendrimer terminal groups than those of the hydrophobic groups located in polymer interior cavities. Molecular modeling also showed polyamine binding with the dendrimer with spermidine-PAMAM more stable than spermine- and BE-333-PAMAM complexes.

In conclusion, we find that synthetic and natural polyamines are capable of binding to different dendrimers. The binding affinity is relatively low to enable them to act as polyamine delivery vehicles, especially polyamine analogues under investigation as cancer chemotherapeutic agents.
